# Promoting advance planning for health care and research among older adults: A randomized controlled trial

**DOI:** 10.1186/1472-6939-13-1

**Published:** 2012-01-05

**Authors:** Gina Bravo, Marcel Arcand, Danièle Blanchette, Anne-Marie Boire-Lavigne, Marie-France Dubois, Maryse Guay, Paule Hottin, Julie Lane, Judith Lauzon, Suzanne Bellemare

**Affiliations:** 1 Department of Community Health Sciences, Faculty of Medicine and Health Sciences, University of Sherbrooke, Sherbrooke, QC, Canada; 2 Research Centre on Aging, University Institute of Geriatrics of Sherbrooke, Sherbrooke, QC, Canada; 3 Department of Family Medicine, Faculty of Medicine and Health Sciences, University of Sherbrooke, Sherbrooke, QC, Canada; 4 Department of Accounting Sciences and Taxation, Faculty of Business Administration, University of Sherbrooke, Sherbrooke, QC, Canada; 5 Department of Psychiatry, Faculty of Medicine and Health Sciences, University of Sherbrooke, Sherbrooke, QC, Canada; 6 Department of Coordination and Academic Affairs, Health and Social Services Centre - University Institute of Geriatrics of Sherbrooke, Sherbrooke, QC, Canada; 7 Member of the Québec Bar, Province of Quebec, Canada

## Abstract

**Background:**

Family members are often required to act as substitute decision-makers when health care or research participation decisions must be made for an incapacitated relative. Yet most families are unable to accurately predict older adult preferences regarding future health care and willingness to engage in research studies. Discussion and documentation of preferences could improve proxies' abilities to decide for their loved ones. This trial assesses the efficacy of an advance planning intervention in improving the accuracy of substitute decision-making and increasing the frequency of documented preferences for health care and research. It also investigates the financial impact on the healthcare system of improving substitute decision-making.

**Methods/Design:**

Dyads (*n *= 240) comprising an older adult and his/her self-selected proxy are randomly allocated to the experimental or control group, after stratification for type of designated proxy and self-report of prior documentation of healthcare preferences. At baseline, clinical and research vignettes are used to elicit older adult preferences and assess the ability of their proxy to predict those preferences. Responses are elicited under four health states, ranging from the subject's current health state to severe dementia. For each state, we estimated the public costs of the healthcare services that would typically be provided to a patient under these scenarios. Experimental dyads are visited at home, twice, by a specially trained facilitator who communicates the dyad-specific results of the concordance assessment, helps older adults convey their wishes to their proxies, and offers assistance in completing a guide entitled *My Preferences *that we designed specifically for that purpose. In between these meetings, experimental dyads attend a group information session about *My Preferences*. Control dyads attend three monthly workshops aimed at promoting healthy behaviors. Concordance assessments are repeated at the end of the intervention and 6 months later to assess improvement in predictive accuracy and cost savings, if any. Copies of completed guides are made at the time of these assessments.

**Discussion:**

This study will determine whether the tested intervention guides proxies in making decisions that concur with those of older adults, motivates the latter to record their wishes in writing, and yields savings for the healthcare system.

**Trial Registration:**

ISRCTN89993391

## Background

### The potential value of advance planning

As the population ages worldwide, growing numbers of older adults develop Alzheimer's disease and related disorders that gradually impair their decision-making capacity [1]. As a result, important health-related decisions end up being made by family members and healthcare providers without direct input from the affected individual [2]. Recent data from the Health and Retirement Study indicate that 40% of decedents require decision making about treatment in the final days of life, of whom 70% lack decision-making capacity [3]. In the context of dementia research, family members are also often solicited for permission to enroll a cognitively-impaired relative in a study with little likelihood of personal benefit [4,5]. Such decision-making is all the more difficult when the wishes of the decisionally-impaired person have never been documented or discussed with family members. Indeed, the scientific literature provides ample evidence that close relatives are unable to accurately predict elderly patients' preferences for care [6] and willingness to engage in clinical studies of varying levels of risk and benefit [7-9].

Advance planning has been widely promoted as a mechanism to guide families toward decisions similar to those an incapacitated relative would have made, had he/she still been able to decide. Advance planning aims at helping people clarify and communicate their values, beliefs and preferences for future care in the event of impaired decisional capacity [10-12]. Led by a trained facilitator, the process involves exploring goals of care in the context of hypothetical illness states. It ideally includes a close family member likely to be involved in decision-making when the patient is incapacitated. Ensuing wishes, called advance directives (ADs), may or may not be conveyed in writing. Written ADs for health care are documents in which capable individuals specify their preferences regarding treatment and/or designate a trusted relative or friend to eventually make decisions for them should they lose the ability to express themselves [13]. When including a section devoted to research [14], written ADs serve the extended purpose of instructing others as to their desire to engage or not in research at times of incapacity [15,16].

Surveys have repeatedly found overwhelming support for ADs, whether communicated in writing or more informally through conversations with families and health professionals [17,18]. Major professional organizations also support advance planning for health care and research [19-21]. Widespread endorsement of ADs is due to their potential to preserve patient autonomy, improve end-of-life medical care, alleviate the burden of substitute decision-making for loved ones, reduce stress, anxiety and depression in surviving relatives, and limit the use of unwanted forms of life-support. ADs may also result in more cost-effective health care by discouraging physicians from resorting to unwanted treatment [17,18,22,23].

Despite widespread professional and public endorsement of ADs for health care, still too few people actually execute them or even discuss future treatment wishes with loved ones or healthcare practitioners [2,18,22,24,25]. The most common reasons include trusting family members to make the right decision when the time comes, the tendency to defer end-of-life planning until facing a life-threatening illness, a lack of knowledge about ADs, and limited access to needed forms [11]. According to our own survey [25], even fewer have voiced their wishes in relation to research involvement. Interestingly, survey participants who had done so were more likely to have conveyed their preferences for care. Accordingly, dedicating a section of AD forms to the person's wishes regarding health research participation could prove effective in increasing the frequency of prospective authorization for research participation [26,27].

### Improving proxy predictions of preferences

Numerous studies have shown that proxy predictions of patient **preferences for health care **are inaccurate [6]. Creative approaches aimed at improving the accuracy of substitute judgment thus need to be developed and evaluated. Most promising approaches include promoting explicit communication between older adults and proxies regarding future medical care, and encouraging patients to document their preferences. The former proposition follows from the repeated observation that prior discussions between patients and proxies about treatment preferences often lead to higher levels of agreement [6,28]. This finding, which stems primarily from cross-sectional studies, needs to be confirmed using stronger designs. At the time of funding, four randomized controlled trials had tested the hypothesis that facilitating discussions about preferences improves the accuracy of surrogate decisions [10,29-31]. A fifth randomized trial was published one year later [32].

Matheis-Kraft & Roberto [29] investigated whether prior discussions of value indicators by older women and their proxies increased the abilities of the latter to make 30 hypothetical healthcare decisions in congruence with those of the women. The experimental dyads (*n *= 30) did not demonstrate statistically higher agreement than their control counterparts (*n *= 30) in 27 of the 30 situations.

The intervention designed by Ditto *et al*. [30] also failed to improve proxies' predictive abilities. Investigators concluded that their findings provide an "unequivocal demonstration of the ineffectiveness of patient-surrogate discussion to enhance the accuracy of substitute judgment". This conclusion may be premature, however, because the intervention was too weak to produce its intended effect. It essentially involved asking patients to complete an AD form in the presence of the surrogate and explain the reasons underlying their choices. Hence patients randomized to the intervention group (*n *= 80) were required to develop a written plan of their wishes, whether or not they had reflected on their personal goals of care and were emotionally ready to commit their preferences in writing. Furthermore, as acknowledged by the authors themselves, the intervention was restricted to a brief, single-session discussion without any explicit educational component on ADs.

Pearlman and collaborators [10] randomized 184 decisionally-competent veteran outpatients who did not have ADs in their hospital record. Experimental subjects were mailed a 52-page advance planning workbook and attended a 30-minute counseling session led by social workers immediately prior to their scheduled appointment with their healthcare providers. Control subjects were mailed the hospital's 8-page AD booklet 5 weeks prior to their office visit. Both books contained blank AD forms to complete. Four months after the index visit, ADs were filed in the medical chart twice as often for the intervention group (48% *vs*. 23%, *p *< 0.001). In addition, healthcare providers were more accurate in predicting the experimental subjects' treatment preferences, values and personal beliefs than for controls (*p *< 0.01 for all three comparisons). Patient-proxy concordance scores did not differ between intervention and control groups for preferences and values, but were higher in the intervention group for personal beliefs (67% *vs*. 56%, *p *= 0.04). Two features of the intervention may explain the better performance of healthcare providers over proxies. Providers were prompted to discuss planning with experimental subjects and had full access to their medical records, including any directives therein. By contrast, no component of the intervention promoted patient-proxy discussion which was entirely left to the patients' discretion. As stated by the authors: "this suggests that specific efforts should be made to include proxies in advance planning discussions with the patients".

Schwartz *et al*. [31] tested whether advance planning facilitated by a trained professional leads to greater understanding by proxies of patients' end-of-life preferences. The control group received a healthcare proxy form to fill out. By contrast, experimental subjects were first handed written information on ADs to be shared with their proxies. Patient-proxy dyads later attended one to two sessions with the facilitator who discussed the benefits and burdens of life-support and documented patient's goals and preferences therefore. Greater concordance was observed among experimental dyads two months post-intervention (Cohen's effect size [ES] = 0.43), a result later confirmed by Gutheil & Heyman [33] (ES = 0.48) using a quasi-experimental design. Effect sizes were not statistically significant due to the small sample sizes (less than 30 dyads per arm). Nonetheless, these studies provide some evidence of the likely benefit of an intervention that combines two strategies previously tested separately: the provision of educational materials on ADs and a facilitated discussion within elderly-proxy dyads about the older adult's values, goals and preferences for end-of-life care.

Lastly, Barrio-Cantalejo *et al*. [32] randomized 171 pairs of patients and their respective proxies to one of three study groups. The control group's answers to the Life Sustaining Preferences Questionnaire (LSPQ) developed by Beland & Froman [34] were compared with their proxies' answers to the same questionnaire. In one intervention group, proxies used the patients' completed AD form to fill in the LSPQ. In the second intervention group, patients and proxies jointly attended two educative sessions with a trained nurse before filling in the LSPQ in separate rooms. The first session was informative and focused on healthcare decisions at the end of life. In the second session, the nurse encouraged each dyad to discuss preferences in different medical scenarios. Accuracy in the discussion group (84.5%) was higher than that observed in the two other study groups (50.34% and 58.3%, respectively). Findings suggest that stimulating dialogue between patients and their proxies about end-of-life preferences improves the accuracy of proxies' predictions much more than isolated use of an AD form. Whether further improvement could be achieved by combining AD forms with discussions remains to be investigated.

No study has yet tested the effect of an intervention in improving family members' abilities to predict their relative's **willingness to engage in clinical research**. Such a study is needed in light of increasing research involving cognitively-impaired older adults [35,36]. Until effective treatments for Alzheimer's disease and related disorders are discovered, clinical research efforts to uncover the causes of such diseases, prevent their onset or halt their progression, will in part depend on the direct participation of cognitively-impaired subjects. However, unlike health care, which is intended to directly benefit the patient, the overriding objective of research is to further knowledge irrespective of any direct benefits the participant may stand to gain. As for health care, most family members are unable to accurately predict their relative's desire to engage in research [7-9]. Nonetheless, researchers turn to them for permission to enrol decisionally-incapacitated prospective subjects [5]. Besides, surveys have repeatedly shown that most people would trust their family to make research-related decisions on their behalf, should the need arise [26,37,38].

To our knowledge, the study by Pearlman *et al*. [10] is the only one that simultaneously tested the effects of patient-proxy advance planning discussions on prediction accuracy and rate of written ADs. Furthermore, we are aware of only one empirical test of ADs for dementia research [15]. As in other studies [29,30], experimental subjects had to complete the directive, which entailed expressing their willingness to enrol or not in five hypothetical research studies. At follow-up, investigators examined the effect of the directive on ease of enrolment decisions in actual studies. Change in proxy accuracy resulting from completing the directive has not been reported. Results are nonetheless relevant to our study as it proved the feasibility of research ADs and willingness of participants to communicate the range of risks they would be willing to assume should they lose decisional capacity and be solicited for research.

## Results from recent systematic reviews

One systematic review has been conducted on **surrogates' predictive accuracy **[6]. It included 16 studies involving 2,595 surrogate-patient pairs asked to make end-of-life treatment decisions in the context of various hypothetical scenarios. Overall, surrogates predicted patients' treatment preferences with 68% accuracy (95% CI: 63% to 72%). Surrogates were more accurate in scenarios involving the patient's current health (79%) than in those involving dementia (58%, *p *< 0.05). Furthermore, one-time informal conversations about patients' preferences were not found to improve surrogate predictive abilities. This review points to the need to find ways to improve surrogates' predictive accuracy, especially under scenarios of altered cognitive states.

We published the latest systematic review of the **effectiveness of interventions in promoting ADs for health care **[39]. Our review includes 55 studies involving 12,691 subjects. Across the 11 randomized trials, the pooled odds ratio for written ADs was 4.0 (95% CI: 1.6-10.4). The intensity of the intervention had the most influence on event rates (*p *< 0.001). Furthermore, the provision of oral information (*p *= 0.039) over multiple sessions (*p *= 0.009) was the main feature of successful interventions. This finding concurs with that of other systematic reviews [40], despite differences in methodological approaches.

In summary, the most recent data available strongly suggest that interventions most likely to be successful in improving surrogates' predictive abilities: involve structured discussions about values and preferences for end-of-life care that go beyond AD completion, are led by trained facilitators, span several sessions, include proxies, and comprise an educational component. Professionally led, multimodal interventions delivered over multiple sessions were also those found most effective in increasing the frequency of written ADs. Our multimodal intervention was designed along these lines.

### The Quebec legal landscape regarding substitute decision-making

In the province of Quebec, Canada, where this trial is conducted, family members are legally authorized to make healthcare decisions for incapable relatives even when not formally appointed to do so through a court ruling (article 15 of the Quebec Civil Code). According to article 12, "a person who gives his consent to or refuses care for another person is bound to act in the sole interest of that person, taking into account, as far as possible, any wishes the latter may have expressed". Yet wishes cannot be taken into account if they have never been communicated to those likely to make substitute decisions. In a recent qualitative study [41], over 70% of family members of a person with advance dementia reported not knowing the healthcare preferences of their relative. Nearly two-thirds of those who had not discussed preferences with their relative wished they could go back to a time when he/she had less cognitive impairment and have the conversation. Furthermore, according to a survey conducted among geriatricians [42], access to written ADs greatly facilitated their discussions about end-of-life care with patients and relatives, even though these directives had not come into effect. Hence, despite not being legally binding in Quebec, prior statements of preferences could be of great assistance to substitute decision-makers and providers when healthcare decisions must be made for incapable patients.

In contrast to health care, consent to non-emergency research for an incapacitated individual can only be given by that person's court-appointed representative (article 21 of the Quebec Civil Code). Yet very few decisionally-incapacitated elders have a court-appointed representative. According to our survey [4], 74.6% of researchers proceed with the consent of a family member, whether legally appointed or not. Moreover, Institutional Review Boards are open to the enrolment in research of incapacitated subjects who do not have a legal representative [4,5]. Thus it is crucial for older adults to inform close family members of the types of research they are willing to take part in, should they be solicited for research after losing decisional capacity.

### The research objectives

The purpose of this trial is to assess the efficacy of a multimodal professionally-led advance planning intervention in 1) improving the accuracy of substitute decision-making (primary outcome) and 2) increasing the frequency of written ADs for health care and research (secondary outcome). Given current knowledge, the tested intervention includes an educational component on ADs and stimulates discussion between an older adult and his/her self-selected proxy with respect to end-of-life care and research participation. The study incorporates a cost analysis to measure public savings that result from improving proxy predictions of preferences.

## Methods/Design

### Design and target population

The efficacy of the proposed intervention in improving prediction accuracy of substitute decision-making is assessed through a single-blind, stratified, randomized controlled trial with prediction accuracy measured at baseline, at the end of the intervention and 6 months later.

The study population is formed of dyads comprising a decisionally-competent older adult and the person he/she would choose to make healthcare decisions on his/her behalf in the event of incompetence (called "the proxy"). Eligible older adults are community-dwelling, French-speaking individuals aged 70 and over who live in the Eastern Townships of the province of Quebec and are likely free of cognitive deficits. Eligible proxies must live in the same region and be fluent in French.

Dyads are randomly assigned to the intervention or control group after stratification for two factors that may influence the success of the intervention: type of designated proxy (spouse *vs*. other) and self-report by the older adult of prior documentation of healthcare preferences (yes *vs*. no). Group allocation is made by reference to computer-generated blocked randomization lists prepared by a team member who has no involvement in delivering the interventions. Random block sizes from 2 to 8 were used within strata to reduce the predictability of group assignment and balance the number of dyads per arm.

### Recruitment process

Prospective subjects are identified from a random list containing the names, sex and addresses of older adults meeting the first four inclusion criteria. The list was extracted from the provincial administrative database containing all beneficiaries of the Quebec universal health insurance plan.

Potential participants are mailed an introductory letter and contacted one week later by the study coordinator who states the purpose of the call, describes how they were chosen and asks for verbal consent to proceed with a short interview. Reasons for not being interviewed (e.g., ineligibility, hearing impairment, refusals) are recorded. Refusers are asked to provide limited descriptive information about themselves for comparison with those who consent to the interview. A 3-item memory test is administered to screen out elders who are likely unable to participate actively in a discussion about their preferences. Older adults excluded because of cognitive impairments are contacted in the following days by a co-investigator to determine how best to assist those in need (e.g., through referral to healthcare providers). Those deemed eligible are invited to participate in the study, which entails designating the person whom they would choose to make healthcare decisions on their behalf in the event of future incompetence. Designated proxies are then contacted by the study coordinator to ascertain their eligibility and own willingness to participate.

Prior to randomization, baseline levels of concordance are established for each eligible and consenting dyad, as follows.

### Baseline concordance assessment

Baseline data are gathered during two face-to-face interviews conducted one week apart by specially trained research nurses who have no involvement in delivering the intervention. The first interview focuses on treatment decisions and the second, on decisions regarding research participation. Interviews are conducted simultaneously, with the elderly person and proxy in separate rooms of the Research Center so that responses are not cross-contaminated.

The first interview begins by asking dyad members to read and sign the informed consent form. Next, the research nurses collect usual socio-demographic data on dyad members, as well as health-related information about the older adult. They then explore various factors that may influence the outcome measures, including personal experience with life-threatening illness, desired level of control in decision-making, and comfort with substitute decision-making. Where relevant, matching questions are administered to the proxies.

The core of the interviews consists of hypothetical clinical and research vignettes specifically designed to elicit older adult preferences and assess the ability of their proxy to predict these preferences. Vignettes are modeled on those developed and validated by us and others [7-9,43-45] in previous research on substitute decision-making. Responses are elicited under four health states: 1) the subject's current health state, 2) as if he/she had sustained a light to moderate stroke, 3) as if he/she had an incurable brain cancer, and 4) as if he/she had severe dementia. The concordance assessment begins by asking older adults to imagine themselves in each of these states and select the answers that best describe their quality of life under these states (from excellent to poor). Then, for each health state, they are asked to indicate whether they would want to receive each of five life-sustaining treatments (antibiotics, CPR, etc.). The same exercise is repeated at the second interview, this time seeking the older adults' willingness to enroll in each of three research protocols (blood sample, supervised exercise program, and oral medication trial). These protocols are designed to cover a broad range of risks and benefits (to the subject or others) in order to elicit an equally broad spectrum of willingness to participate. Older adults are also asked to select one out of four goals of care (from palliative care to intensive care) for different sudden health events (pneumonia, hemorrhagic shock, and severe head trauma). Health events were chosen to capture a wide range of conditions varying in severity, nature of impairment, and prognosis. To ensure understanding, health states, treatment modalities, research protocols and critical events are described briefly and simply by the research nurse using written materials handed out during training. Using identical response scales, proxies are asked to choose the decision they think the older adult will make in each situation (substituted judgment) or, if unable to do so, to make a decision based on the older adult's best interests. Concordance assessments were pre-tested on three potential dyads and subsequently revised for clarity. Results are compiled as soon as the interviews are completed, and communicated to experimental dyads at the start of the intervention.

### Trial interventions

#### Intervention in the experimental group

The proposed intervention was designed from literature reviews [6,39,40] and recent experimentations [31,33]. It integrates multiple methods to increase learning [46] and uses teaching tools adapted to normal aging changes [47]. The intervention consists of 2-hour meetings, once monthly, over three months. The duration of the intervention follows from the need for an incremental process that enables older adults to articulate preferences at their own pace and gradually integrate information on the benefits and limitations of ADs [28]. Furthermore, we expect repeated encounters to foster trusting relationships between dyad members and research personnel that are conducive to discussions on issues as sensitive as end-of-life care. The first and third meetings take place in the home of the older adult or proxy, a strategy likely to counter attrition [31]. The second meeting is an information session on ADs for groups of dyads.

Home meetings are led by trained social workers with extensive experience working with elders. The first visit begins with two thought-provoking vignettes that highlight the importance of communicating one's wishes about future medical care and research participation. The use of vignettes illustrating real-life experience has been shown to increase learning in elderly populations [47]. After eliciting reactions to the vignettes and exploring related issues, the facilitator summarizes the dyad-specific results of the concordance assessment and invites dyad members to discuss the reasons underlying their choices. Discussions seek to help older adults describe what makes their life worth living in specific, functional terms [48] and articulate their wishes, personal values and beliefs, so as to guide proxies in making decisions for them. Throughout the intervention, the social worker catalyzes discussion so that a shared understanding gradually develops between the older adult and his/her proxy.

The AD information session focuses on a guide entitled *My Preferences *that we designed for expressing wishes regarding future health care and research involvement. *My Preferences *also invites the signer to designate substitute decision-makers should he/she become incompetent. It was adapted from an AD form developed by Molloy and collaborators [49] for the *Let Me Decide *(LMD) project. The LMD form has been successfully implemented in nursing homes and the community [50-53]. *My Preferences *was also inspired by documents developed by others for the same purpose [44,54,55]. Adaptation involved describing Quebec's legislation regarding substitute decision-making and adding a section for expressing desire to engage or not in studies of varying levels of risk and benefit.

*My Preferences *contains information on the purposes and uses of ADs. It addresses their practical limitations, including the impossibility of anticipating all future medical events [56-58]. The importance of reviewing ADs periodically is underscored given that preferences may change with the occurrence of significant life events, as predicted by the response shift theory [22,59,60]. More importantly, *My Preferences *emphasizes the difference between healthcare and research ADs so that subjects understand that research aims primarily at advancing knowledge for the benefit of future patients.

*My Preferences *was designed to be attractive to and effective with older adults [46,47,61]. It requires no more than 30 minutes to read and includes features such as:

■ paper with a matte surface,

■ contrasted background and text,

■ font size no smaller than 13 points,

■ short sentences using words as simple as possible,

■ glossary with medical and technical term definitions,

■ main idea of each paragraph highlighted in a box,

■ many illustrations,

■ space to write down instructions.

Before its use in the study, *My Preferences *was presented to 10 persons and subsequently revised for clarity.

The learning environment for the information session is also adapted for the elderly: a large, well-lit room allowing participants to move around easily, small group setting with chairs in a U shape layout to encourage interaction and discussion [47,62,63]. Various effective teaching tools are used throughout the information session [47,48,62-65], including:

■ a quiz to introduce and help assimilate new information,

■ a PowerPoint presentation in bright colors with large text,

■ videos showing people sharing their experiences (e.g., a person expressing difficulties due to not knowing the wishes of a loved one, a physician explaining the importance of advance planning for health care) or explaining how to use *My Preferences *to convey their wishes to family members,

■ activities encouraging participants to express their own point of view and to start completing *My Preferences *which then serves as an external memory aid to help overcome deficits in memory processes that are frequent among older adults.

At the third and final meeting, the social worker checks subjects' understanding of the information delivered at the group session, explains any terms in *My Preferences *that are still unfamiliar to the participants, and assists older adults in filling one out if desired. Those who elect to complete *My Preferences *at this stage are advised to inform family members and designated substitute decision-makers of its location and content. The social worker then requests a copy for analysis purposes. Content analyses of completed guides will allow us to determine whether the older adult designated a substitute decision-maker and/or expressed preferences regarding health care and research participation. Lastly, the social worker explores participants' satisfaction with the process and collects any suggestions they might have on how to improve the intervention.

#### Intervention in the control group

Approximately once monthly over three months, dyads assigned to the control group are invited to attend a 2-hour health education workshop on healthy lifestyle and preventive practices. The three workshops are adapted from a health education program aimed at improving self-care skills that has been effective with elderly people [66]. The number of meetings between dyads and the research team is identical across groups. However, experimental dyads meet individually and in subgroups whereas controls always convene in subgroups. Health education workshops are given by experienced nurses and allow for interaction within and between dyads. Once the study is complete, the AD information session given to experimental dyads will be offered to controls.

### Primary and secondary outcome measures

Prediction accuracy of substitute decision-making is the primary outcome and rate of completed guides, the secondary outcome. Concordance is the most important outcome of advance planning activities. However, we consider important to also measure the rate of documented preferences as these could raise less controversy in the event of incompetence than oral instructions given only to a single family member.

Concordance assessments are repeated at the end of the intervention (first posttest) and 6 months later (second posttest), according to the same procedure as at baseline. However, the first posttest uses new sets of treatments, sudden health events and research protocols to counter recall bias. Older adults' decisions under these various scenarios, and their proxies' predictions concerning those decisions, are recorded by the same research nurses who interviewed them at baseline. The second posttest will allow us to determine whether the positive effect of the intervention on prediction accuracy, if any, is maintained for at least six months. Copies of completed *My Preferences *are made at the first posttest and, for those who have yet to complete one, at the second posttest. Allocating six months to fill out *My Preferences *gives participants extra time to decide whether they want to commit directives in writing. A longer interval carries the risk of losing too many participants and no longer permits attributing the observed effect to the intervention, as opposed to more recent events that might have occurred in the meantime. Evidence regarding the impact of the intervention on the rate of completed guides will be based on written documents rather than self-report as the latter was found to overestimate true completion rates [39].

### Cost Analysis

To measure the intervention's financial impact, we will use the goals of care chosen by the older adults and their proxies at baseline and at the second posttest for the various health events. These goals of care will have been transformed into costs previously. The differential cost between the decisions made at baseline by the older adults in the experimental group and those made by their proxies for all scenarios will be deducted from the differential cost at posttest 2. To establish the net effect, the results will be compared with those of the control group calculated with the same method.

Three steps are required to cost each of the possible healthcare scenarios (health states × goals of care × health events): 1) identifying the healthcare resources required; 2) measuring the amount of resources previously identified; and 3) costing each resource [67,68].

As in previous studies [53,69,70], to identify the resources to be costed, we considered the health care and services that could be influenced by the preferences of the older adults or their proxies and could thus generate different costs. Therefore, we will value the healthcare services delivered essentially by the public healthcare system: hospital stays, nursing and assisted care, general and specialized medical care, ambulance transportation and other direct or indirect treatment-related costs. We will disregard healthcare services provided by family members on a volunteer basis.

To determine the number of resources required, we asked three physicians to create a treatment profile for each scenario, which were later validated from existing care protocols [71-78]. Relying on their expertise and experience, the physicians established a list of healthcare services based on the most frequent situations in their practice for cases similar to the various scenarios provided. These treatment profiles therefore combine a number of healthcare services to make up a "typical" treatment for each scenario in the study.

Costing each identified resource is the last step. Although the cost of the resources should normally be their opportunity cost [67,79], we will in fact be using the market cost, which is considered to be equivalent [79,80]. Therefore, the value of medical and nursing care will be based on the salary earned by a physician or health professional [67,81] while materials required for treatments will be valued at their purchase price. When it will not be deemed pertinent to determine the value of a resource according to the amount spent, because that amount is no longer representative of its value (as with infrastructures, for example), we will use the market value [67,81]. Various data (government statistics, financial reports from healthcare institutions, etc.) will be used to establish resource costs, which will be set in 2010 dollars. Where appropriate, a government indexation rate will be applied [67].

### Compliance and losses to follow-up

Workshop attendance by control dyads is monitored and will be used to interpret study findings, as will attendance of the information session by experimental dyads. Social workers record the number of meetings held with dyads that later drop out. Dyads that withdraw their participation are reached by phone, once, and invited to return to the Research Center for a follow-up assessment. Posttests are conducted at home when most convenient for participants. Those refusing are asked whether they have committed their preferences in writing and, if so, to provide a copy of *My Preferences *completed. Concordance data are not available from dyads that refuse further involvement as such data cannot be collected over the phone.

### Potential limitations

People who agree to participate may differ to some extent from those who refuse. In particular, participants may be more favorable towards ADs - especially research ADs - than non-participants. Information gathered during the first telephone contact with prospective subjects will provide some data to compare participants with those who decline participation.

Participants are not explicitly informed of the study hypotheses. However, they cannot be blinded completely to group assignment. This design feature may exert some influence on their decision to complete *My Preferences*. However, it is unlikely to affect the primary outcome (prediction accuracy of substitute decisions) since dyad members are tested in separate rooms with vignettes that differ in part from those used at baseline. The use of control dyads partially controls for the Hawthorne effect on outcome measures. Furthermore, to ensure that participants who choose to express their preferences in writing understand the purposes and uses of written ADs, all participants are administered a brief knowledge questionnaire at the last posttest.

Every effort is made to keep research nurses blind to group assignment, including instructing participants not to reveal their group assignment. Unblinding may occur in some cases but this should not affect results since concordance is assessed by two nurses in separate rooms. While nurses may inadvertently discover that the dyad was exposed to the intervention (or not), this knowledge cannot impact concordance as they are unaware of the answers given by the other dyad member. Contamination is highly unlikely since controls do not meet their experimental counterparts and the consent form does not detail the tested intervention. Co-interventions are also unlikely given the relatively short duration of the intervention and scarcity of AD promotional efforts in the province of Quebec.

Last but not least, hypothetical vignettes do not generate the emotional reactions that characterize real-life situations. In addition, preferences may change as a result of declining health. Despite these drawbacks, the use of hypothetical scenarios in eliciting preferences for times of incapacity is common practice in advance care planning and research on substitute decision-making. More importantly, the intervention raises participants' awareness of the importance of updating ADs regularly.

### Statistical analyses

Persons who decline participation or drop out will be compared to trial participants with parametric or non-parametric two-sample tests (depending on the distribution of the data) and results used to judge the scope of study findings. The same tests will be used to compare participants' baseline characteristics across study groups. Potential confounders and compliance indices will be candidates for adjustment when testing the impact of the intervention on the outcome measures [82]. All analyses will be based on allocation to the intervention or control group (i.e. by intention-to-treat). To account for losses to follow-up, analyses will be conducted in duplicate, first restricting the sample to the dyads for which we have complete follow-up data, then using a conservative approach by which dyads that do not provide such data are assumed not to have documented preferences and their baseline concordance levels are used for imputation [83].

The primary analysis will involve calculating composite scores of agreement across health states for each dyad. Calculations will generate a total concordance score as well as sub-scores derived from restricting summations to the clinical or research vignettes. Growth-curve modeling implemented with SAS PROC MIXED will be used to compare composite scores across time points and groups, an approach that accounts for the within-dyad correlation over time and accommodates missing values [84,85]. To further elucidate the impact of the intervention, secondary analyses will involve applying the same statistical procedure to the concordance sub-scores. A multivariable logistic model will be used to compare group-specific completion rates of *My Preferences *6 months post-intervention. The financial impact of the intervention will be investigated with Student's *t *test for independent samples or its non-parametric equivalent.

### Sample size

We are aiming for 86 dyads per group at study completion, which would provide 80% power to detect a standardized difference of 0.43 in overall prediction accuracy between the study groups (calculation based on the *t *statistic, two-sided α = 0.05, nQuery Advisor 6.0). The expected standardized difference was derived from Schwartz *et al*. [31] whose study resembles our own in many respects. The tested interventions are of comparable intensity and duration. Both include an AD educational component and devote a central role to proxies. Moreover, in both cases, prediction accuracy is measured with a composite index derived from combining proxy's ability to predict the older adult's preferences under various hypothetical scenarios.

**Figure 1:**
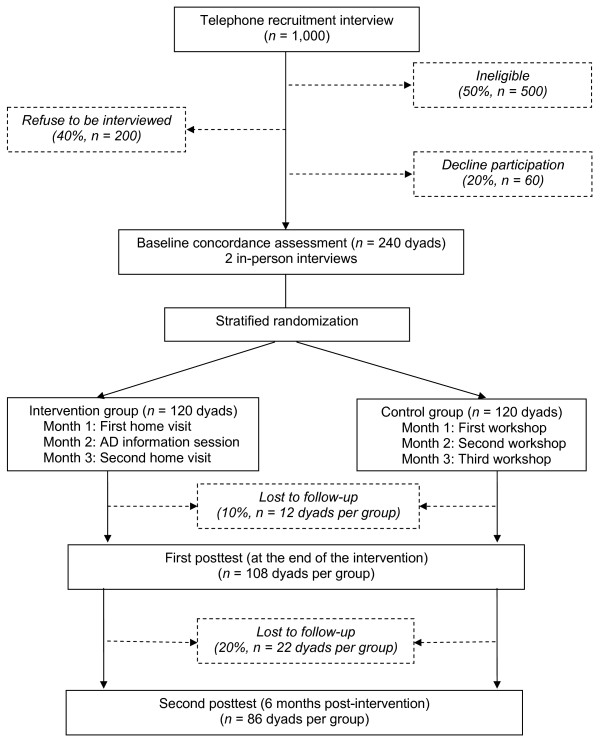
**Study subjects flow chart (with estimated sample sizes in parentheses)**.

Accounting for a drop-out rate of 10% at posttest 1 and 20% at posttest 2, 240 dyads will be randomized. As indicated in Figure 1, we started the recruitment process with a random list of 1,000 individuals who fulfill our first four inclusion criteria. This number should allow us to reach the target sample size, further accounting for sampled individuals who will be found ineligible (e.g., institutionalized or deceased, no longer reside in the study area, no proxy meeting study criteria), refuse to be interviewed, or decline study participation.

The above calculations assume that we will compare groups on posttest mean concordance scores as this allowed us to use the best available estimate of the likely effect of the intervention. However, our design involves repeated measurements of concordance over time that will be analyzed by growth-curve modeling, a more sophisticated approach that effectively uses all the available time points to estimate individual trajectories. Determination of suitable sample sizes for multilevel analyses is complex, in part because explicit formulas have not yet been derived for general models. However, simulations by Mass & Hox [86] show that between 50 and 100 units per group are needed to justify claims as to the absence of bias in standard error estimates. As shown in Figure 1, projected sample sizes are 120 units per group at baseline, 108 at posttest 1 and 86 at study completion. Since all data will contribute to some extent in estimating the model parameters, our approach to sample size estimation is conservative and protects against an underpowered study.

Lastly, as shown in Table 1, 86 dyads per group also provide sufficient power to detect a meaningful difference between groups for the secondary outcome.

**Table 1:** Power to detect the intervention effect on documented preferences, according to three sources of information (*n *= 86 dyads per group, two-sided α = 0.05)

Source	Odds ratio (95% CI)	Event rate in the Control group	Event rate in the Experimental group	Power
Bravo *et al*. [39]	4.0 (1.6 - 10.4)	0.12*		95%
Patel *et al*. [87]	3.7 (1.5 - 9.4)	0.12*		92%
Pearlman *et al*. [10]		0.23	0.48	93%

* Derived from 13 RCTs included in our systematic review (mean = 0.12, sd = 0.182, range: 0 to 0.56) and applied to Patel *et al*. who did not report the mean event rate across control groups.

### Ethical considerations

The study protocol and materials have been approved by our Institutional Review Board. This study carries little risk to participants. Some people may feel uncomfortable discussing end-of-life treatment preferences or envisioning themselves or their loved ones in a state of mental incapacity. End-of-life issues and decisional incapacity are sensitive matters for most people. However, the vast majority welcome opportunities to discuss them and actually wish that health professionals would raise the subject. In any event, study participants will be duly informed of their right to withdraw from the study, at any time and for any reason, without prejudice.

## Discussion

Study results will expand current knowledge on advance planning by determining whether professionally-led discussions of preferences improve substitute decision-making and yield cost savings. Findings will also inform future public policy and legislation changes by supplying population-based estimates of the proportion of older adults who choose to document their wishes regarding future health care and research participation. Such data are essential to determine whether the practice of accepting consent from families in treatment settings (even lacking prior expressed wishes) should carry over to research venues [88].

If found effective, subsequent studies could aim at extending the accessibility of the intervention beyond study participants. Pamphlets underlying the importance of intra-familial discussions about end-of-life care and research participation could be distributed at various locations (e.g., waiting rooms at health clinics, seniors' clubs, residential care facilities) and include a quiz designed to assess the ability of family members to predict a relative's wishes. During medical checkups, physicians could probe patients' interest in advance planning, give copies of *My Preferences *to those interested and refer them to a social worker in charge of delivering the advance planning intervention. However, we must first ensure that the proposed intervention does in fact significantly enhance substitute decision-making abilities.

### Current study status

Enrollment began in April 2011.

## Abbreviations

AD and ADs: advance directive(s); ES: effect size; CI: confidence interval; sd: standard deviation; CPR: cardiopulmonary resuscitation.

## Competing interests

The authors declare that they have no competing interests.

## Authors' contributions

GB is the principal investigator and lead author. GB, MA, AMBL, MFD, MG, PH, and JLau were members of the initial research team and contributed to the development and writing of the study protocol. JLau left the research team in April 2011. JLan and DB joined the team in November 2009 with specific responsibilities in implementing the experimental intervention (JLan) or designing and conducting the cost analysis (DB). All team members were actively involved in designing *My Preferences *and concordance assessments, with marked input from MA, AMBL and PH. Activities offered to the control group are coordinated by MG. Data entry and analysis is under the responsibility of MFD, who also supervises the randomization process. SB is the study coordinator. All authors read and approved the study protocol.

## Pre-publication history

The pre-publication history for this paper can be accessed here:

http://www.biomedcentral.com/1472-6939/13/1/prepub
